# The Relationship between Childhood Abuse and Suicidal Ideation among Chinese College Students: The Mediating Role of Core Self-Evaluation and Negative Emotions

**DOI:** 10.3390/bs14020083

**Published:** 2024-01-24

**Authors:** Zhaoxia Pan, Dajun Zhang, Xiaohua Bian, Hongye Li

**Affiliations:** 1 Faculty of Education Science, Zhengzhou Normal University, Zhengzhou 466000, China; bxh1999@zznu.edu.cn (X.B.); lhy061061@stu.zznu.edu.cn (H.L.); 2 Centre for Mental Health Education and Research, Faculty of Psychology, Southwest University, Chongqing 400715, China; zhangdj@swu.edu.cn

**Keywords:** childhood abuse, suicidal ideation, adolescents, core self-evaluation, negative emotion

## Abstract

Childhood abuse is a significant risk factor for suicidal ideation. However, the underlying mediation mechanism necessitates further exploration. This study investigated the mediating role of core self-evaluation and negative emotions in the relationship between childhood abuse and suicide ideation in young adults. A sample of 3103 college students from 11 universities across 8 provinces in China was analyzed. Childhood abuse, core self-evaluation, negative emotions, and suicidal ideation were assessed using the Childhood Trauma Questionnaire (CTQ-CF), Core Self-Evaluation Scale, Affect Scale, and Beck Suicidal Ideation Scale (BSI-CV), respectively. Data analysis was conducted using SPSS 19.0 and SPSS Macro Process. We found that high scores for childhood abuse were associated with elevated levels of suicidal ideation, whereas low scores for core self-evaluation were closely linked to heightened levels of negative emotions and suicidal ideation. Furthermore, core self-evaluation and negative emotions mediated the relationship between childhood abuse and suicidal ideation through three significant paths. The results demonstrate that childhood abuse can directly impact suicidal ideation in young adulthood and indirectly influence suicidal ideation by affecting core self-evaluation and negative emotions. They suggest that addressing core self-evaluation and negative emotions in individuals who have experienced childhood abuse may help prevent or treat suicidal ideation.

## 1. Introduction

Suicidal ideation refers to an individual thinking of and planning to end their own life. It is a precursor of suicidal behavior and an important indicator in the field of mental health. In recent years, the problem of college student suicidal ideation has attracted worldwide attention. According to the World Health Organization (WHO), suicide has become the second leading cause of death among young people aged 15 to 29, an age group that covers the majority of college students [1]. Internationally, a study covering 48 countries found that about 10.6% of college students reported having serious suicidal ideation [2]. In the United States, according to a survey by the American College Health Association (ACHA), 7.4% of college students seriously considered suicide at least once in the past 12 months, whereas 1.9% of them reported actually having made a suicide plan during the same time period [3]. Suicide is also one of the leading causes of death among young people in China. A survey of college students showed that the incidence of suicidal ideation varied by school and region but the average incidence was 9.2% [4]. These data suggest that suicidal ideation is a problem among college students that cannot be ignored. It not only poses a threat to individual life safety but also has a profound impact on families and society [5]. The consequences of suicidal behavior cause family members to experience grief and trauma while also placing an economic burden on society [6]. The cost of suicide and suicide-related behavior to the global economy is estimated to be in the billions of dollars per year [5]. In addition, the presence of suicidal ideation is often associated with other mental health problems, such as depression, anxiety, and substance abuse, which, if left untreated, can lead to decreased academic performance, broken relationships, and an overall decline in the quality of life. In summary, the issue of suicidal ideation among college students is not only an important public health challenge but also an urgent problem in the field of mental health. Given the intricate nature and urgency of this issue, a thorough examination of the risk factors associated with suicidal ideation and its underlying mechanisms among college students is imperative. However, current research in this domain remains in its infancy, particularly regarding understanding the complex processes leading to suicidal ideation among college students. This situation somewhat restricts the formulation and implementation of effective prevention and intervention measures. Therefore, this study aims to systematically and deeply explore this field to reveal the underlying mechanisms driving college students’ suicidal ideation. Through this examination, we hope to offer fresh perspectives and approaches to addressing this pressing issue, ultimately fostering progress in related prevention and intervention efforts.

The field of developmental psychopathology offers a foundational framework for examining the determinants of suicidal ideation among college students. Studies indicate that maltreated children who fail to reach developmental milestones commensurate with their age encounter greater challenges in subsequent developmental stages and exhibit an elevated susceptibility to depressive symptoms and suicidal thoughts [7,8]. The cumulative situational risk hypothesis posits that an accumulation of adverse experiences in early life escalates the potential for developmental risks to compound, thereby intensifying the likelihood of suicidal thoughts [9]. A critical factor in predicting suicidal thoughts under adverse conditions is the experience of childhood maltreatment. This term encompasses actions by caregivers responsible for a child’s upbringing, supervision, and development that may inflict or have the potential to inflict harm on the child’s health, survival, growth, development, or dignity. It spans a spectrum of maltreatment, including physical or emotional abuse, sexual abuse, neglect, and economic exploitation [10]. Such experiences of abuse during childhood have been linked to increased aggression in later life, which can be directed outwardly towards others or inwardly in the form of self-injury, self-harm, or suicide [11]. Empirical findings also suggest that individuals with a history of childhood abuse struggle to reconcile residual childhood conflicts, rendering them more susceptible to suicidal ideation and behaviors [12]. Consequently, we posit Hypothesis 1 (H1): There is a significant positive correlation between childhood maltreatment and the emergence of suicidal ideation among Chinese college students.

The impact of early-life maltreatment on the emergence of suicidal ideation during young adulthood is a complex issue. The cognitive coherence optimization model of suicide posits that self-perception plays a pivotal role in the genesis of suicidal ideation, offering insights into this phenomenon [13]. Recurrent abusive behaviors by caregivers during childhood convey detrimental messages to children, insinuating a lack of worth, inherent defects, unlovability, peril, superfluity, and futility. Such abusive encounters precipitate the assimilation of a negative self-image, culminating in diminished self-esteem, identity, and efficacy. Research indicates a direct influence of childhood maltreatment on one’s fundamental self-assessment [14,15]. Core self-evaluation represents the most rudimentary judgment and appraisal of one’s capabilities and worth, encompassing self-esteem, locus of control, neuroticism, and general self-efficacy [16]. Empirical evidence suggests a linkage between early-life abuse and the quartet of core self-evaluation. Experiences of maltreatment correlate positively with neuroticism [17] and inversely with self-esteem [18] and self-efficacy [19]. Additionally, individuals subjected to abuse often exhibit a propensity for an external locus of control [20]. Consequently, we advance Hypothesis 2 (H2): Childhood abuse precipitates suicidal ideation via the intermediary influence of core self-evaluation.

Negative affects encompass a spectrum of subjective distress and aversive experiences [21], indicative of enduring personal emotional variances. Findings have revealed that individuals with lower core self-evaluation are prone to heightened negative affect, including stress [22], depression [23], and loneliness [24], as well as fear and anxiety [25]. The cognitive vulnerability model of depression, corroborated by empirical research, demonstrates that negative cognitions can precipitate depression, with excessively adverse self-perceptions potentially triggering a cascade of negative emotions and, ultimately, suicidal ideation. Thus, we propose Hypothesis 3 (H3): Childhood abuse impairs core self-evaluation and elevates negative affect, culminating in suicidal ideation.

Childhood abuse is a salient contributor to the onset of emotional disturbances [20]. Exposure to physical, psychological, and sexual abuse heightens the risk of developing pronounced depressive symptoms, with a dose–response relationship observed between abuse severity and symptomatology [26,27]. The emotional hypothesis of suicide posits that negative emotional states such as depression and anxiety are direct precursors to suicidal ideation [28]. Therefore, we introduce Hypothesis 4 (H4): Childhood abuse engenders negative emotions, which in turn lead to the formation of suicidal thoughts.

Drawing upon the cognitive coherence optimization model of suicide and the emotional hypothesis of suicide, this investigation delves into the mediating roles of core self-evaluation and negative affect in the relationship between childhood abuse and suicidal ideation. This study aims to elucidate potential determinants and pathways influencing adolescent suicidal ideation, thereby furnishing a theoretical framework for the prophylaxis and intervention in youths’ suicidal conduct.

## 2. Materials and Methods

### 2.1. Participants

Participants in the study were recruited from a population of college students aged 16 to 24 years, with exclusion criteria including a history of significant mental health disorders, as evidenced by hospitalization. The questionnaire was meticulously crafted to include a specific inquiry: “Have you ever been hospitalized due to a major mental health condition?” This question was designed to screen for participants with a history of severe mental illnesses that could potentially confound the study’s findings.

A cohort of 3103 college students, comprising 2280 females with an age distribution of 16 to 24 years (mean age = 20.05 years, standard deviation = 1.47 years), was assembled from a stratified random sample of classes across 11 universities located in eight Chinese provinces: Henan, Ningxia, Hainan, Liaoning, Inner Mongolia, Hubei, Jilin, and Beijing. The participants were gathered in relaxing classroom settings to complete the questionnaires, which were presented in Chinese. Under the guidance of trained investigators, the students responded to the items included within the questionnaires. Anonymity was maintained, as the participants were not asked to provide their names. Data from 90 individuals were discarded due to incompleteness or anomalous responses. The remaining students finished the questionnaire within an average time of 10 min. Subsequently, each participant was remunerated with CNY 5 for their involvement. Ethical approval for the research was granted by the institutional review board of the hosting university, and written consent was secured from all participants.

### 2.2. Measures

Childhood abuse. The Childhood Trauma Questionnaire (CTQ-CF), developed by Bernstein [8], served as the instrument to assess the extent of childhood maltreatment among participants. The psychometric properties of the Chinese adaptation, including its reliability and validity, have been established in a collegiate sample [29]. This instrument encompasses 28 items systematically organized into five subscales: emotional abuse, physical abuse, sexual abuse, emotional neglect, and physical neglect. Responses are measured on a 5-point Likert scale, ranging from 1 (never) to 5 (always). Scores for each subscale range from 5 to 25, culminating in a composite score of between 25 and 125 [8,30], with three additional items dedicated to assessing validity. Higher aggregate scores indicate more severe experiences of childhood maltreatment [30]. The Cronbach’s alpha for the scale in the current research was reported as 0.931, denoting excellent internal consistency.

Suicidal ideation. The assessment of suicidal ideation was conducted utilizing the Beck Suicidal Ideation Scale (BSI), a widely recognized instrument in both clinical settings and research domains [31,32]. The BSI’s Chinese adaptation, known as the BSI-CV, demonstrated robust reliability and validity in studies involving university populations [33,34]. This scale comprises 19 items, each rated on a three-tier scale, designed to gauge the intensity of suicidal thoughts either over the past week or during the peak of such ideation. The scale bifurcates into two dimensions: The first five items pertain to the ideation of suicide, whereas the subsequent 14 items pertain to suicidal actions. This study employed only the ideation component to explore the depth of suicidal thoughts during the most critical phase. The highest score for this dimension is 15. Scores correlated positively with the severity of suicidal ideation and the associated risk of suicide. The reliability of the scale, as indicated by a Cronbach’s alpha coefficient, was found to be 0.885 in this study.

Core self-evaluation. The assessment of participants’ core self-evaluation was conducted utilizing the Core Self-Evaluation Scale [16]. The instrument’s Chinese adaptation, refined by Du, Zhang, and Zhao [34], has undergone rigorous reliability and validity evaluations within a collegiate demographic [35,36]. Comprising 10 items, the scale encapsulates both affirmative and critical self-assessments. Responses are gauged on a quintuple-point continuum, anchored by “strongly disagree” at one end and “strongly agree” at the other. An aggregate score on the higher end reflects a more pronounced core self-evaluation. The highest score on this scale is 20. In the context of this investigation, the scale demonstrated a Cronbach’s alpha coefficient of 0.858, indicating high internal consistency.

Negative emotions. The assessment of participants’ negative affective states was conducted utilizing the Affect Scale developed by Bradburn [37]. The psychometric properties of the scale’s Chinese adaptation have been established in a collegiate sample [38]. Comprising both positive and negative affect dimensions, the scale is designed to evaluate emotional states and equilibrium. However, the present research focused exclusively on the negative affect dimension to gauge the intensity of participants’ negative emotional experiences. This subscale consists of five items, with responses recorded as binary “yes” or “no” options, translating to scores of 1 or 0, respectively. The aggregate of these scores yielded an overall negative emotion index, where higher totals signified elevated levels of negative affect. The highest score for this dimension is 5. The internal consistency of the negative emotion subscale in this investigation was confirmed, with a Cronbach’s alpha coefficient of 0.704.

### 2.3. Data Analysis

Initially, to address the potential for common method bias, which is inherent in studies that rely solely on self-report data, we employed Harman’s single-factor test at the outset of our analysis. This test is designed to identify whether a single factor accounts for the majority of the variance in the data, which could indicate a bias towards self-reporting. By implementing this test, we aimed to minimize the influence of common method variance on our findings.

Following the initial assessment, we proceeded with a comprehensive variance analysis, descriptive statistics compilation, and an examination of the interrelationships among the variables. This step was crucial for understanding the underlying structure of the data and ensuring that our subsequent analyses were grounded in a solid empirical foundation.

In the next phase of our research, we focused on the mediating effects of core self-evaluations and negative affectivity. To investigate these mediating roles, we employed the PROCESS macro (Model 6) as outlined by Hayes [39]. This macro is specifically tailored for the analysis of mediation models, enabling us to explore the complex pathways between variables with precision. We included the gender and academic year of the college student participants in our models, as these factors are known to influence psychological outcomes. By estimating a 95% confidence interval for a random sample of 5000 subjects, we aimed to enhance the robustness of our results and ensure that our findings are representative of the broader population.

## 3. Results

### 3.1. Common Method Deviation Test

Given that the dataset was exclusively derived from self-administered surveys, there was a potential for common method bias. Heeding the recommendations of Zhou and Long [40], we implemented both procedural and statistical controls to mitigate this bias. Procedurally, during the survey design phase, we designed some reverse-scoring items and ensured the anonymity of the participants. Statistically, we employed Harman’s single-factor test to assess the common method variance [41]. An exploratory factor analysis was conducted on all survey items, unveiling eight distinct factors with eigenvalues exceeding 1.0. Notably, the principal factor explained a mere 28.51% of the total variance, falling well below the 40% threshold, thereby confirming that common method bias was not a significant issue in our research [42].

### 3.2. Preliminary Analysis

The descriptive statistical analysis results show that the mean childhood abuse score was 35.74, the mean suicidal ideation score was 7.07, the mean core self-evaluation score was 34.35, and the mean negative emotion score was 1.59.

The preliminary analysis revealed a significant positive association between childhood abuse and suicidal ideation (r = 0.345, *p* < 0.01) (H1). Additionally, there was a negative correlation between childhood abuse and core self-evaluation (r = −0.300, *p* < 0.01), as well as core self-evaluation and suicidal ideation (r = −0.345, *p* < 0.01). Furthermore, the analysis indicated a positive relationship between negative emotions and both childhood abuse and suicidal ideation (r = 0.239 to 0.287, *p* < 0.01), whereas negative affect showed a negative association with core self-evaluation (r = −0.497, *p* < 0.01). For more detailed information, please refer to Table 1.

### 3.3. Testing for Mediating Effects

As presented in Table 2, the results indicate that childhood abuse significantly and positively predicted suicidal ideation (β = 0.064, *p* < 0.0001) while negatively predicting core self-evaluation (β = −0.563, *p* < 0.0001) and positively predicting negative emotions (β = 0.053, *p* < 0.0001). Moreover, core self-evaluation significantly predicted negative emotions (β = −0.110, *p* < 0.0001) and suicidal ideation (β = −0.082, *p* < 0.0001).

**Figure 1:**
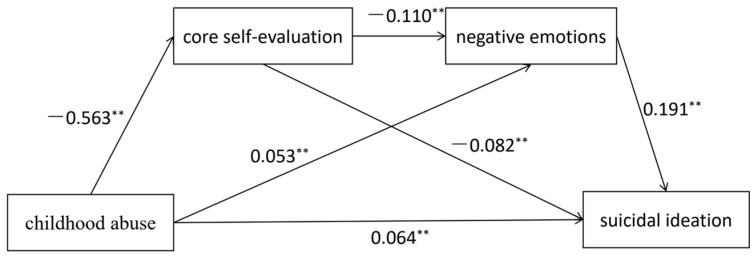
Mechanism of childhood abuse on suicidal ideation. ** *p* < 0.01.

The mediation analysis, as illustrated in Table 3 and Figure 1, revealed that the bootstrap 95% confidence interval for the total indirect effect did not encompass 0. This indicates that core self-evaluation and negative emotions played a mediating role in the relationship between childhood abuse and suicidal ideation. The mediating effect size was estimated to be 0.0171, accounting for 26.62% of the total effect. The mediating effect was composed of three indirect effects: (H2) indirect effect 1 (0.0116): childhood abuse → core self-evaluation → suicidal ideation, (H3) indirect effect 2 (0.0032): childhood abuse → core self-evaluation → negative emotions → suicidal ideation, and (H4) indirect effect 3 (0.0023): childhood abuse → negative emotions → suicidal ideation. These indirect effects accounted for 18.06%, 5.01%, and 3.55% of the total effects, respectively. The bootstrap 95% confidence interval for each indirect effect did not include 0, indicating that all three indirect effects were statistically significant.

## 4. Discussion

The results of this investigation lend credence to Hypothesis 1, aligning with the extant body of literature [43] in positing that experiences of childhood abuse are a salient harbinger of suicidal ideation in young adults. This study invokes the pathological model of psychological development, which posits that individuals engage in a process of positive adaptation, cultivating motivation, attitudes, coping mechanisms, emotions, and interpersonal skills; yet, the scourge of traumatic experiences in one’s formative years can severely hinder the maturation of these critical faculties. Consequently, individuals who have endured trauma may find themselves bereft of the requisite adaptability and resources during pivotal developmental junctures, thus compelling them to seek solace in maladaptive behaviors such as self-harm or suicide as a means to navigate life’s vicissitudes [44]. In essence, suicide may be conceptualized as a maladaptive compensatory strategy that arises in the wake of insufficient psychological development attributable to early-life trauma.

Furthermore, the findings bolster Hypothesis 2 and the cognitive aggregation hypothesis of suicidality. Hovens, Giltay, van Hemert, and Penninx [45] similarly discerned that core self-evaluation mediates the relationship between childhood maltreatment and its deleterious aftermath. Victims of childhood abuse often internalize negative external judgments into their self-assessment [46], culminating in a diminished sense of self-worth and perceived control. This internalization process results in a compromised core self-evaluation [47]. When such individuals encounter subsequent adversities, including further abuse, bullying, or discrimination, they are predisposed to non-adaptive cognitive responses, characterized by internalized negative attitudes and beliefs. In processing external stimuli, these individuals are prone to a subjective belief that they will be met with rejection and unfair treatment, leading to psychological and behavioral dysregulation and, ultimately, suicidal ideation. These findings also resonate with Bronfenbrenner’s ecological systems theory, suggesting that distal adverse developmental experiences and proximal irrational self-perceptions synergistically precipitate suicidal ideation in adolescents. The insights gleaned from this study contribute to elucidating the etiology of adolescent suicidal ideation.

The study also uncovered that childhood abuse leads to suicidal ideation through a “core self-evaluation–negative emotion”-mediated pathway, corroborating Hypothesis 3. This may be attributed to the fact that individuals with low core self-evaluations often feel powerless to influence their own performance, which in turn propels them towards escapism rather than proactive problem-solving. This behavioral pattern engenders negative emotions [48,49], thereby establishing a continuous mediating effect between core self-evaluation and negative emotions in the context of suicidal ideation. By integrating the cognitive convergence hypothesis and the emotional hypothesis of suicide, this study elucidates how adverse childhood experiences precipitate cognitive and emotional turmoil, rendering adolescents unable to evade the detrimental impacts, potentially culminating in dire outcomes such as suicidal ideation or behavior.

Moreover, the research identified negative emotions as a mediating factor in the nexus between childhood abuse and adolescent suicidal ideation, lending support to Hypothesis 4. Although numerous studies have delved into the mediating role of discrete emotions—such as depression, anxiety, loneliness, feelings of inferiority, and shyness—in the relationship between childhood abuse and suicidal ideation, it is important to note that many negative emotional experiences are interconnected [50]. Consequently, debates and conceptual overlaps among fundamental emotional categories have exacerbated the instability of research findings [51]. This study, therefore, has adopted a more nuanced conceptual framework to explore the mediating role of negative trait emotions in the relationship between childhood abuse and adolescent suicidal ideation, thereby fostering a more profound comprehension of the pivotal role emotions play in the genesis of adolescent suicidal ideation.

In conclusion, the results confirm our theoretical hypothesis that childhood abuse, as an important risk factor for suicidal ideation, affects college students’ suicidal ideation through three clear pathways involving core self-evaluation, emotional status, and a combination of the two. First of all, childhood abuse has a direct impact on college students’ core self-evaluation, which may cause individuals to question their own value and the meaning of their existence and then trigger suicidal ideation. Secondly, this experience of abuse also affects the daily emotional state of college students, making them more prone to ongoing emotional distress and psychological stress, which increases the risk of suicidal ideation. In addition, we found that abuse experiences further affected individuals’ emotional states by affecting their core self-evaluations, a chain reaction that further exacerbated suicidal ideation. The findings of this study substantiate the childhood abuse–core self-evaluation–negative emotions–suicidal ideation model and comprehensively unveil the mechanisms by which childhood abuse influences college students’ suicidal ideation from both cognitive and emotional standpoints. This research provides novel empirical backing through an in-depth examination of the determinants and mechanisms underpinning college students’ suicidal ideation, thereby enriching the theoretical discourse on the subject. Additionally, the outcomes of this study offer fresh perspectives on the prevention and intervention of adolescent suicidal ideation. Given that young adulthood is a period marked by rapid psychological and physiological development, often accompanied by the dual pressures of academic and emotional challenges, it is imperative to shield young individuals from abuse while concurrently addressing and rectifying maladaptive self-cognitions to avert suicidal ideation stemming from diminishing core self-evaluations. An intervention program targeting adolescent suicidal ideation, predicated on core self-evaluation, could be devised based on the findings of this study, thus presenting a novel approach to the prevention and intervention of suicidal ideation and behavior among adolescents.

Nevertheless, this study is not without its limitations. Its cross-sectional design precludes the establishment of causality. Future research could employ longitudinal designs and experimental methodologies to ascertain and validate causal relationships. Additionally, the study’s participant pool was limited to college students, not exclusively adolescents, thereby necessitating further verification to determine whether the research model and conclusions are generalizable to younger adolescent cohorts. Lastly, the reliance on self-report measures may have introduced bias due to social desirability effects. Subsequent studies should endeavor to utilize more objective data collection techniques.

## 5. Conclusions

This study has elucidated a significant association between experiences of childhood abuse and the emergence of suicidal ideation, suggesting that core self-evaluations and negative affect may serve as mediators within this relationship. Individuals subjected to abuse during their formative years are prone to developing distorted perceptions of self, which can amplify their experience of adverse emotions and, consequently, elevate the risk of contemplating suicide. It is imperative for mental health professionals to thoroughly assess core self-evaluations and the spectrum of negative emotions in young adults with a history of childhood abuse. Furthermore, a comprehensive approach to the assessment, prevention, and intervention of suicidal ideation must incorporate considerations of past childhood abuse to ensure effective treatment outcomes.

## Figures and Tables

**Table 1:** Descriptive statistics, standard deviations, and correlations of the main study variables.

Variable	M ± SD	1	2	3	4	5
1 Gender	1.73 ± 0.44	1				
2 Grade	2.34 ± 1.08	0.009	1			
3 Childhood abuse	35.74 ± 13.76	−0.192 **	0.115 **	1		
4 Core self-evaluation	34.35 ± 6.43	−0.065 **	−0.04 *	−0.300 **	1	
5 Negative affect	1.59 ± 1.52	0.012	0.075 **	0.239 **	−0.497 **	1
6 Suicidal ideation	7.07 ± 2.56	0.006	0.043 **	0.345 **	−0.345 **	0.287 **

Note. *n* = 3103; gender was dummy coded (1 = male, 2 = female); * *p* < 0.05, ** *p* < 0.01.

**Table 2:** Regression analysis of variable relationship in the model.

Dependent Variable	Independent Variable	*R*	*R* ^2^	*F*	*Β*	*t*
Suicidal ideation	Childhood abuse	0.345	0.345	0.345	0.064	20.44 ***
Core self-rating	Childhood abuse	0.287	0.083	279.13	−0.563	−16.71 ***
Negative emotion	Childhood abuse	0.509	0.259	543.17	0.053	7.105 ***
	Core self-rating				−0.110	−28.786 ***
Suicidal ideation	Childhood abuse	0.459	0.210	275.11	0.224	17.01 ***
	Core self-rating				−0.082	−10.98 ***
	Negative emotion				0.191	6.125 ***

Note: *** *p* < 0.001.

**Table 3:** Mediation effect analysis.

	Effect	Boot SE	Boot LLCI	Boot ULCI	Relative Indirect Effect
Total indirect effect	0.0171	0.0015	0.0144	0.0203	26.62%
Indirect effect 1	0.0116	0.0013	0.0092	0.0143	18.06%
Indirect effect 2	0.0032	0.0006	0.0021	0.0045	5.01%
Indirect effect 3	0.0023	0.0006	0.0013	0.0036	3.55%

## Data Availability

Upon a justified request, the corresponding author can provide the dataset used in this analysis and the images evaluated in the photograph-rating task.
